# Lipidomics and RNA-Seq Study of Lipid Regulation in *Aphis gossypii* parasitized by *Lysiphlebia japonica*

**DOI:** 10.1038/s41598-017-01546-1

**Published:** 2017-05-02

**Authors:** Gao XueKe, Zhang Shuai, Luo JunYu, Lü LiMin, Zhang LiJuan, Cui JinJie

**Affiliations:** Institute of Cotton Research, Chinese Academy of Agricultural Sciences/State Key Laboratory of Cotton Biology, Anyang, Henan 455000 China

## Abstract

The cotton–melon aphid, *Aphis gossypii* Glover, is a major insect pest worldwide. *Lysiphlebia japonica* (Ashmead) is an obligate parasitic wasp of *A*. *gossypii*, and has the ability to regulate lipid metabolism of the cotton-melon aphid. Lipids are known to play critical roles in energy homeostasis, membrane structure, and signaling. However, the parasitoid genes that regulate fat metabolism and lipid composition in aphids are not known. 34 glycerolipids and 248 glycerophospholipids were identified in this study. We have shown that a 3-day parasitism of aphids can induce significant changes in the content and acyl chain composition of triacylglycerols (TAGs) and subspecies composition of glycerophospholipids content and acyl chains. It also upregulate the expression of several genes involved in triacylglycerol synthesis and glycerophospholipid metabolism. Pathway analysis showed that a higher expression of genes involved in the tricarboxylic acid cycle and glycolysis pathways may contribute to TAGs synthesis in parasitized aphids. Interestingly, the higher expression of genes in the sphingomyelin pathway and reduced sphingomyelin content may be related to the reproductive ability of *A*. *gossypii*. We provide a comprehensive resource describing the molecular signature of parasitized *A*. *gossypii* particularly the changes associated with the lipid metabolism and discuss the biological and ecological significance of this change.

## Introduction

The cotton-melon aphid, *Aphis gossypii* Glover, is an important agricultural pest, and is a major pest of cotton in China^1, 2^. It can cause serious damage to cotton seedlings during the early growing season, by sucking on host plants. The natural enemy of cotton aphid is the wasp, *Lysiphlebia japonica* (Ashmead), which is the predominant parasitoid of cotton–melon aphids and is a dominant species in the fields of northern China. This wasp can effectively control cotton aphid populations in early summer^3, 4^ thus projecting a promising future in the control of aphids by biological control strategy.

It has been reported that parasitoids can modify host metabolism such that the host nutrition sustains the wasp’s growth than the host themselves^5–9^. Particularly, parasitoids are known to regulate the hosts’ fatty acid composition, which is a major factor influencing host suitability to parasitoids^10^. For example, *Pseudaletia separate* parasitized by *Euplectrus separatend* for 3 to 8 days had significantly higher fat content in the hemolymph than non-parasitized *P*. *separate* ^11^. A number of studies have shown that parasitoids regulate the metabolism in fat body to increase the content of triacylglycerol and fatty acids in the hemolymph^12–14^. Triglycerides (TAGs) in the fat body cells are the main energy source for insects at different developmental stages^15^. Other lipids such as phospholipids (PLs) function as structural components of cell membranes. The fluctuation of the composition of these lipids provide new insights into the physiological state of insects^16–19^.

Parasitoids have been reported to affect lipid synthesis in cotton–melon aphids^20, 21^. However, it is not known how these changes in lipid synthesis affect the cellular and physiological processes of the cotton–melon aphid. In this study, we investigated the effects of *L*. *japonica* parasitism on the lipid metabolism of *A*. *gossypii*. RNA-Seq combined with ultra-high performance liquid chromatography (UHPLC) coupled with hybrid quadrupole time-of-flight mass spectrometry (Q-TOF-MS) based metabolomics was applied to determine the effects of parasitism on the gene expression and probe content-dependent alterations in the metabolic profiles of parasitized and non-parasitized control aphids. Our results showed that parasitism resulted in marked changes in the lipid metabolism, and composition and remodeling of glycerolipids and glycerophospholipids. These results provide novel insights into the lipid metabolism of parasitized aphids and will enable further characterization of the lipid synthesis pathway and its relationship with the tricarboxylic acid (TCA) cycle and glycolysis.

## Materials and Methods

### Insects and Parasitoids

Mummified *A*. *gossypii* were collected from a cotton field in the Institute of Cotton Research at the Chinese Academy of Agricultural Sciences (36°5′34.8″N, 114°31′47.19″E). A single population of *A*. *gossypii* that reproduced by parthenogenesis was used in the study. Aphids were reared on cotton leaves at 26 ± 1 °C, 65 ± 5% relative humidity and a 14:10 h light:dark photoperiod. To rear *L*. *japonica*, they were placed together with the aphids and maintained at 24 ± 1 °C, 75 ± 5% relative humidity and a 14:10 h light:dark photoperiod. To generate parasitized aphids, third instar cotton-melon aphids were exposed to wasps until parasitism was observed. Both parasitized and non-parasitized aphids were reared for 3 days on cotton leaves in incubators. Aphids were collected and dissected under the microscope to separate the parasitized from non-parasitized aphids and to remove the wasp larvae.

### Lipid extraction

We maintained two groups of aphids; non-parasitized and parasitized (after removing *L*. *japonica*) with six biological replicates per group with 12 samples/group. To extract lipid metabolites from tissues, each sample was weighed and 20 mg of the tissue was homogenized in ball mill after addition of 5 balls and 100 μL water, at 4 °C and 30 Hz for 3 min. The homogenate was then transferred to a glass tube, mixed with 400 μL CH_2_Cl_2_:MeOH (2:1, v/v) and vortexed for 30 s. The mixture was then centrifuged for 15 min at 2500 rpm and the organic layer (~200 μL) was transferred to a clean glass vial and evaporated to dryness; the dried extract was reconstituted in 100 μL CH_2_Cl_2_:MeOH (1:1, v/v) and used in UHPLC-QTOFMS analyses.

### Lipid Analysis–Mass Spectroscopy

The following reagents were used in the experiments: ACN (Honeywell, Japan), HCOONH_4_ (Thermo, USA), and IPA (Thermo, USA). The TripleTOF mass spectrometer was used for its ability to acquire MS/MS spectra on an information-dependent basis (IDA) during an LC/MS experiment. In this mode, the acquisition software (Analyst TF 1.7, AB Sciex) continuously evaluates the full scan survey MS data as it collects and triggers the acquisition of MS/MS spectra depending on preselected criteria. In each cycle, 6 precursor ions whose intensity greater were higher than 100 were chosen for fragmentation at collision energy (CE) of 35 V (15 MS/MS events with product ion accumulation time of 50 msec each). ESI source conditions were set as following: Ion source gas 1 as 60, Ion source gas 2 as 60, Curtain gas as 30, source temperature 550 °C, Ion Spray Voltage Floating (ISVF) 5500 V or −4500 V in positive or negative modes, respectively. Lipids were tested on UHPLC-qTOFMS (Agilent 1290 UHPLC + AB Triple TOF 6600+), Chromatographic column was from Kinetex (Phenomenex, USA; 1.7 u C18 100 A 100 × 2.1 mm). The TripleTOF mass spectrometer was used for its ability to acquire MS/MS spectra on an information-dependent basis (IDA) during an LC/MS experiment. In this mode, the acquisition software (Analyst TF 1.7, AB Sciex) continuously evaluates the full scan survey MS data as it collects and triggers the acquisition of MS/MS spectra depending on preselected criteria. Mobile phase settings were: A: 10 mM HCOONH_4_ in ACN/H_2_O (v/v, 6:4), B: 10 mM HCOONH_4_ in ACN/IPA (v/v, 1:9); Program: 0:00 min, 400 µL/min, 60% A, 40% B; 9:00 min, 400 µL/min, 0% A, 100% B; 10:00 min, 400 µL/min, 0% A, 100% B; 10:20 min, 400 µL/min, 60% A, 40% B; 13:00 min, 400 µL/min, 60% A, 40% B. MS parameter settings were: GS1: 60 psi; GS2: 60 psi; CUR: 30 psi; TEM: 600 °C; ISVF: 5000 V; TOF Masses (Da): Min = 200.0000, Max = 1200.0000.

### Statistical Analysis

Raw data were converted to mzXML format using ProteoWizard, and further analyzed by XCMS. Metabolites were identified by accurate mass search (<30 ppm) and MS/MS spectral match using an in-house standard MS/MS library.

Peaks were detected and metabolites could be left through interquartile range denoising method, and then, missing values of raw data were filled up by half of the minimum value. In addition, overall normalization method was employed in this data analysis. The resulting three-dimensional data containing the peak number, sample name, and normalized peak area were fed to the SIMCA14 software package (Umetrics, Umea, Sweden) for principal component analysis (PCA) and orthogonal projections to latent structures-discriminate analysis (OPLS-DA). In order to obtain a higher level of group separation and to better understand the variables responsible for classification, supervised OPLS-DA was applied. Then, parameters for the classification of R^2^Y and Q^2^Y were obtained from the software and checked for stability and good to fitness prediction. To estimate robustness and the predictive ability of our model we used the 7-fold cross validation method with permutations. R^2^ and Q^2^ intercept values were obtained after 200 permutations. Low values of the Q^2^ intercept show the robustness of the models, and thus indicate a low risk for over fitting and reliability. Based on OPLS-DA, a loading plot was constructed, and it showed the contribution of variables to differences between the groups (Figure S2). It also showed the important variables that were situated far away from the origin. However, the loading plot is complex because of many variables.

Heatmap (R package, Version 3.2.3) was used for a more intuitive analysis of the differences among these components. The relative difference (percent) between the two groups was estimated using the Hodges-Lehmann estimator, X axis stand relative difference (%). To refine the analysis, variable importance projection (VIP) was the first principal component measured. VIP values exceeding 1.0 were first selected as changed metabolites. The remaining variables were then evaluated by Student’s *t*-test (*p* > 0.05) and those without significant differences were discarded^22, 23^. Moreover, we used fold change as another criterion to assess differences in the levels of compounds (fold change >1.5 or <0.5 and *p* < 0.05). In addition, commercial databases, including NIST (http://www.nist.gov/index.html) and KEGG (http://www.genome.jp/kegg/) were used for qualitative analysis and to search for the metabolites in the lipid biosynthesis pathway. The metabolights number is MTBLS410.

### Extraction of RNA and RNA-Seq data analysis

Total RNA was extracted from both parasitized (after removing *L*. *japonica*) and non-parasitized aphids using TRIZOL reagent (Invitrogen, Carlsbad, CA, USA) following the manufacturer’s instructions. Each of the parasitized and non-parasitized groups had three biological replicates. Illumina sequencing of the samples was performed at the Beijing Genomics Institute (Shenzhen, China). After filtering the raw reads by removing the adapter, poly-N and low quality sequences, we used Trinity to perform *de novo* assembly of the clean reads. Then, TAGicl was used to cluster transcripts to obtain unigenes. TAGicl was applied to each sample individually and repeatedly to obtain the final unigenes for downstream analyses. Unigenes larger than 150 bp were first aligned by BLASTN to the NCBI-Nt database (e-value < 10^−5^) and by BLASTX to protein databases including NCBI-Nr, Swiss-Prot, KEGG and COG (e-value < 10^−5^) to retrieve proteins along with their functional annotations. DEseq2, based on the negative binomial distribution, was performed as described previously^24^. Differential expression was determined using a cutoff significance level of false discovery rate (FDR) < 0.05. Functional enrichment analysis was performed with HOMER^25^ using pathways related to metabolism from the KEGG database annotation^26–28^. Expression abundance analysis of the unigenes was calculated based on the reads per kilobase per million mapped reads (RPKM) method, which eliminated the influence of different gene lengths and sequencing discrepancies while calculating expression abundance. The clean reads and computationally assembled sequences have been submitted to the NCBI/SRA database and NCBI/TSA repository. The ArrayExpress accession number is MTAB-5228.

## Results

### Mass Spectra and Pattern Recognition Analysis of Lipids Extracts

Each positive mode and negative mode received 12 samples and 5 QC samples. Among these, 2056 peaks were positive, and 3757 peaks were negative. Mass spectra of lipid extracts from the control and parasitized aphids were dominated by signals of mass lipids. The resonance of lipids was assigned based on the literature and results from the lipid map. Mass spectra were mainly composed of various phospholipids (PLs) and triacylglycerols (TAGs). A complete list of the lipidomics data is provided in Table S1.

**Figure 1:**
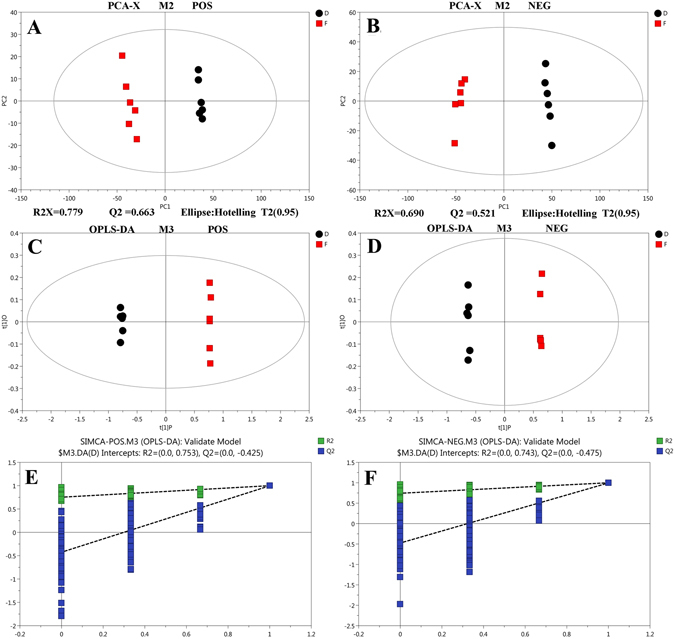
Score plot of the PCA (**A**,**B**) and OPLS-DA (**C**,**D**) models applied to parasitized and control aphids (POS and NEG). (**A**,**B**) Score plot of PCA model obtained from D and F (POS and NEG). (**C**,**D**) Score plot of OPLS-DA model obtained from D and F (POS and NEG). ^a^(**E**,**F**) Score plot of OPLS-DA model obtained from D and F (POS and NEG). ^a^Two hundred permutations were performed, and the resulting R2 and Q2 values were plotted. Green square, R2; Blue square, Q2. The green line represents the regression line for R2 and the blue line for Q2.

We identified a total of 34 different TAGs and 248 PLs (POS and NEG) from the D and F samples. To evaluate whether parasitism affects lipid synthesis and composition, 70 kinds of phospholipids were selected for further analysis. In the lipidomics analysis, six independent pairwise comparisons were performed to eliminate false positives and negatives, to view only the robust alterations in lipid composition after parasitism. Principle component analysis (PCA) was initially applied to the spectra to visualize inherent clustering between control and parasitized classes. As shown in Fig. 1(A,B), unsupervised PCA revealed noticeable separation between Non-parasitized and parasitized. There was an obvious difference in the lipid composition between twogroups. In order to obtain a higher level of group separation and for a better understanding of the variables responsible for classification, supervised OPLS-DA was applied. Characteristics of the models generated are summarized in Table 1. Good separation of the lipid extracts between Non-parasitized and parasitized groups were achieved as shown in the OPLS-DA scores plots (Fig. 1C–F).

**Table 1:** Summary for the OPLS-DA Model-Based Discrimination between parasitized and non-parasitized (parasitized VS non-parasitized) aphids Using Cross-Validation.

Model	Type	A	N	R^2^X (cum)	R^2^Y (cum)	Q^2^ (cum)	Title
M3	OPLS-DA	1 + 1 + 0	12	0.936	1	0.997	POS
M3	OPLS-DA	1 + 1 + 0	12	0.858	1	0.995	NEG

R^2^X and R^2^Y values indicate the total number of variations in the X and Y matrix explained by the model, respectively. Q^2^ represents the predictability of the models and relates to its statistical validity.

### Parasitism Drastically Changes the Expression of Genes Related to Lipid Metabolism

**Figure 2:**
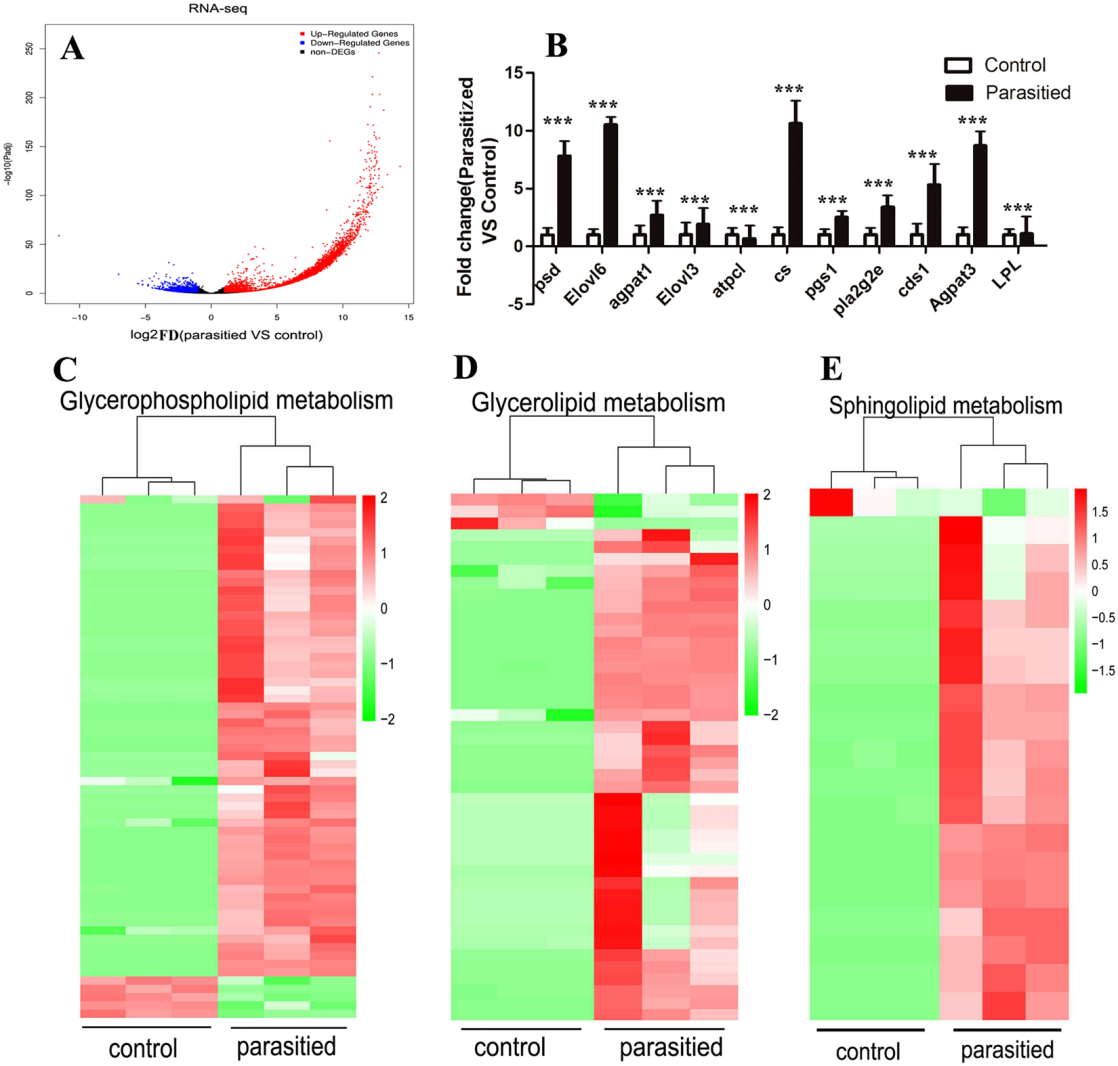
Parasitization Induces Expression of Genes Involved in Lipid Metabolism. Total RNA obtained from both parasitized and non-parasitized control aphids was used for RNA-seq. Differential mRNA expression between parasitized and control samples was determined by false discovery rate (FDR). (**A**) Log2 fold changes in RefSeq genes in parasitized and control aphids, and the corresponding significance values are displayed as-log10 (FDR). X axis represents −log10 transformed significance. Y axis represents log2 transformed fold change. Red dots represent up-regulated DEGs. Blue dots represent down-regulated DEGs. Black dots indicate non-DEGs. (**B**) Relative expression levels of up-regulated and down-regulated genes from the RNA-Seq data in the parasitized aphids compared to the control aphids. Data represent the mean of six samples. ***FDR < 0.001, control vs parasitized aphids. (**C**–**E**) Heatmap of genes in the glycerophospholipid metabolism (**C**), glycerolipid metabolism (**D**) and sphingomyelin metabolism (**E**) in the parasitized and control aphids. The log10 expression values for each sample were clustered. Scale bars on the top right of each heatmap represent the degree of gene expression with red showing high expression and green indicating low expression.

Lipidomics changed significantly in parasitized aphids and was evidenced as changes in the expression of genes involved in the lipid-associated pathway between parasitized and non-parasitized insects. Both parasitized (after removing *L*. *japonica*) and non-parasitized aphids were tested to map the transcriptional changes in *A*. *gossypii* in response to parasitism. The *A*. *gossypii* sequences revealed substantial (48.65%) matches with *Acyrthosiphon pisum*. Analysis of the unigenes resulted in the identification of differentially expressed genes (DEGs) using DEseq2. Using a significance level of *p* < 0.05, we found a total of 14,455 DEGs among which 13,015 were up-regulated and 1,440 were down-regulated (Fig. 2A).

We then processed the DEGs to obtain their functional classification using the Gene Ontology (GO) and KEGG databases. We found that some DEGs in the metabolic pathways and signal transduction were significantly enriched, which included glycerolipid metabolism, glycerophospholipid metabolism, and sphingomyelin (Fig. 2). Several genes involved in these pathways had increased expression in parasitized aphids and included the fatty acyl chain elongase. Particularly, *Elovl3* (the gene encoding the main enzyme involved in elongation of saturated and monounsaturated C18-C22 fatty acid substrates^29^) and *Elovl6* (elongation of very long chain fatty acids protein 6) (Fig. 2B) displayed significantly higher expression levels after parasitism. We also detected a marked increase in the expression of *Psd* (phosphatidylserine decarboxylase), *Agpat1*/*3* (1-acyl-sn-glycerol-3-phosphate acyltransferase), Pgs1 (phosphatidylglycerophosphate synthase 1), *Pla2g2e* (secre tory phospholipase A2) and *Cds1* (CDP-diacylglycerol synthase 1), which are involved in the glycerophospholipid metabolism pathway (Fig. 2B). Up-regulated genes related to the glycerolipid metabolism included *LPL* (lipoprotein lipase) and *cs* (citrate synthase) while *atpcl* (adenosine triphosphate (ATP) citrate lyase) was significantly down-regulated after exposure to parasitoids (Fig. 2B) indicating an inhibitory feedback circuit. In addition, genes related to the MAPK signaling pathway, and antigen processing and presentation also increased significantly after parasitism by *L*. *japonica* (Figure S1). Together, these results indicate that a 3-day parasitism induces extensive changes in the transcriptome of *A*. *gossypii* when compared to non-parasitized aphids. These changes may contribute significantly to the growth and development of *L*. *japonica* and may eventually lead to the death of *A*. *gossypii*. Different expressed genes in some related pathway were showed in Table S2.

### Parasitism Affects the Composition of Fatty Acyl Chains but not the Lipid Class of TAG in *A*. *gossypii*

**Figure 3:**
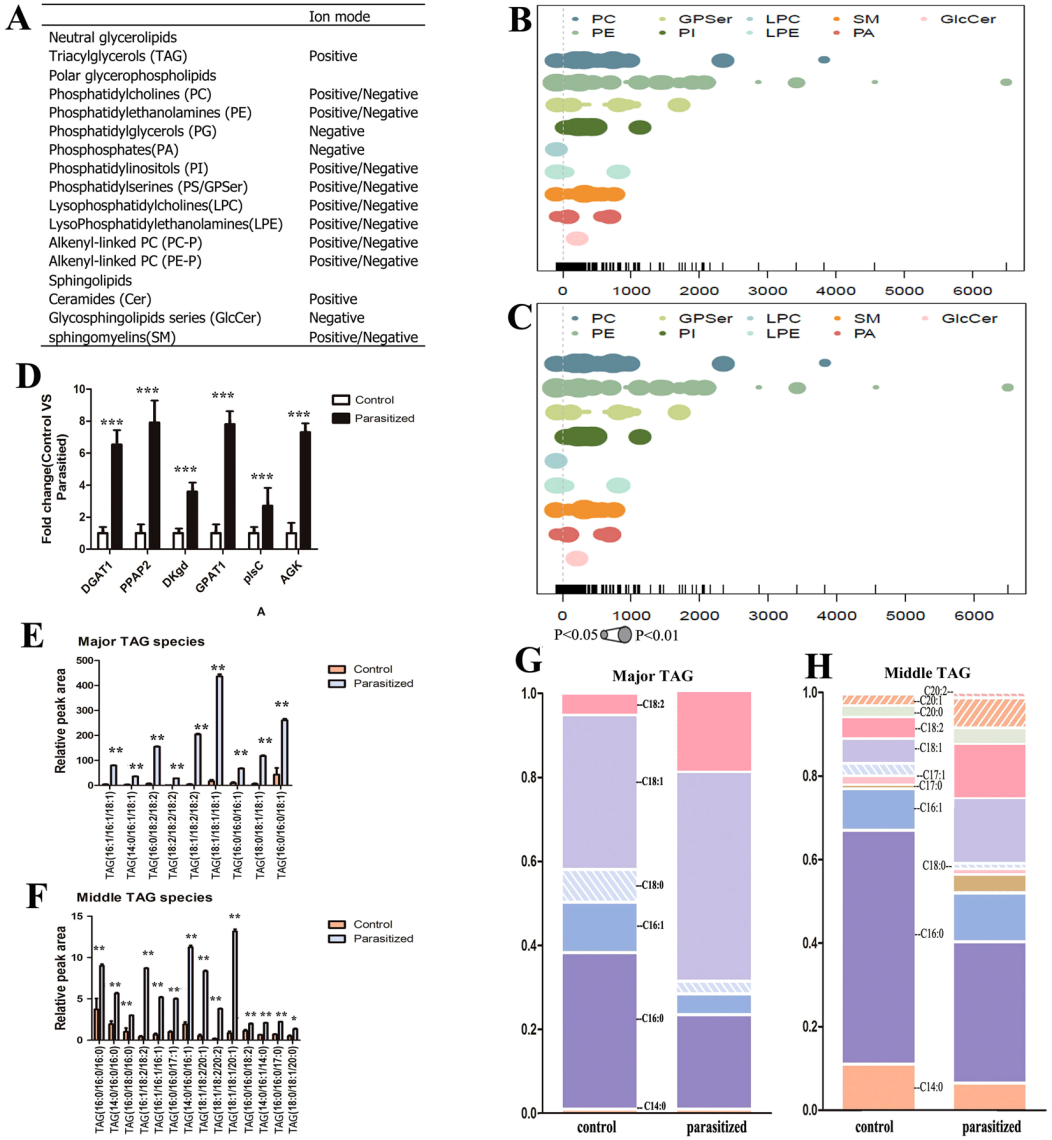
Changes in the Lipid Composition and Fatty acyl chains of triglycerides (TAG) in parasitized aphids. Lipid classes identified in lipidomics experiments and their abbreviations used in this paper. (**B**,**C**) Differences in the relative percentage of all quantified lipid species between parasitized and control aphids (POS and NEG). Each spot represents a lipid species, the spot size indicates significance, and the different colors represent different lipid species. n = 6/group. (**D**) Relative expression levels of selected genes involved in glycerolipid metabolism. Data are means ± SEM; n = 3/group. ***FDR < 0.001. (**E**) The relative peak area of quantified major TAG species in parasitized and control aphids. Data are means ± SEM; n = 6/group. **p < 0.01. (**F**) The relative peak area of quantified middle TAG species in parasitized and control aphids. Data are means ± SEM; n = 6/group. *p < 0.05, **p < 0.01. (**G**,**H**) Changes in glycerolipid base chemistry and fatty acyl chains of major TAG (**G**) and middle TAG (**H**) in parasitized and control aphids. Data are means ± SEMαn = 6/group).

**Figure 4:**
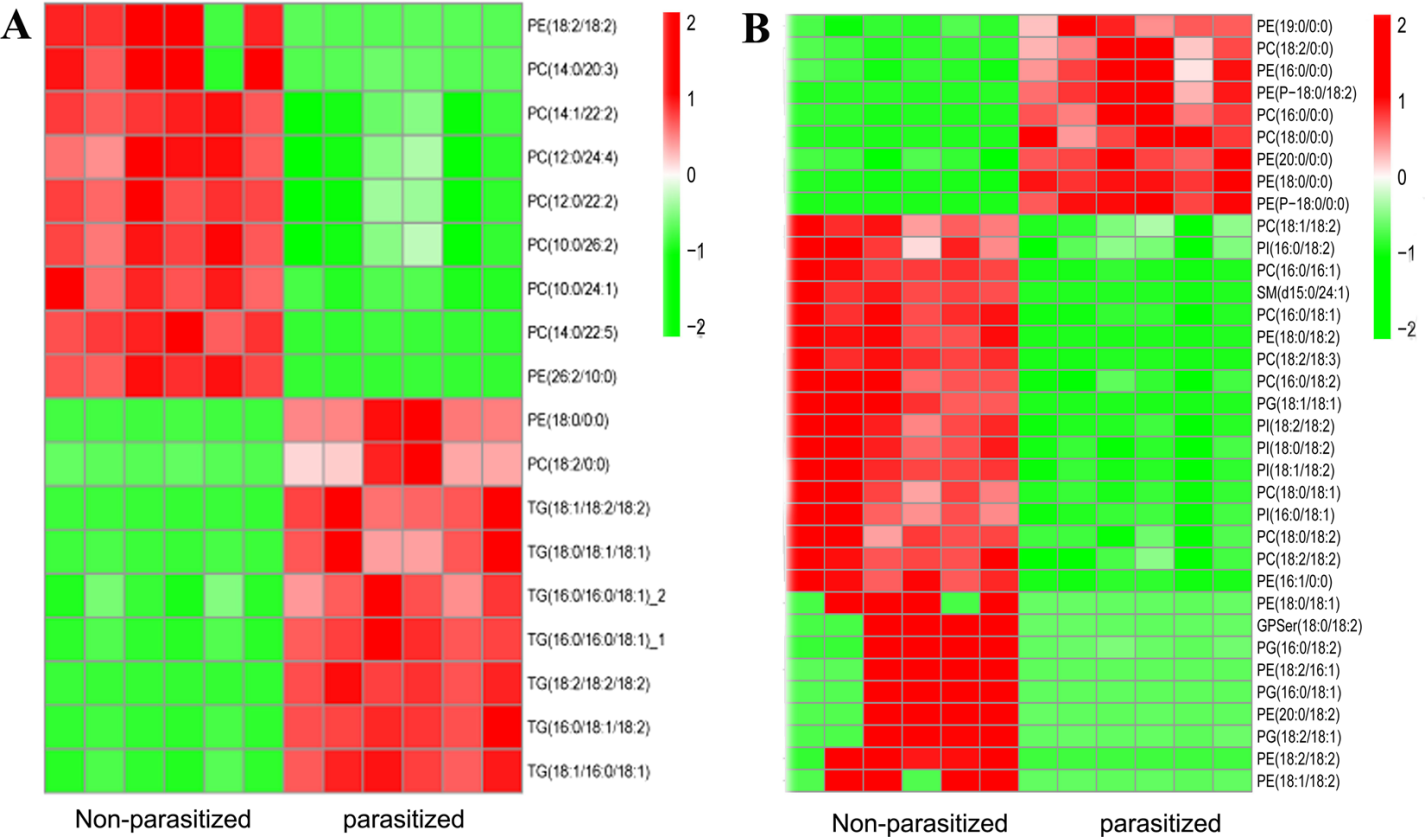
Heatmaps of the integration of intermatrix Lipids correlation between D vs F (**A**- POS, **B** - NEG). Heat maps were applied to display the quantitative expression of lipids with a red color gradient representing high expression and a green color gradient representing low expression. Lipid synthesis metabolites with significant changes in the non-parasitized aphids and parasitized aphids groups from UHPLC-Q-TOF-MS was analyzed in the heatmap (VIP > 1, *p* value < 0.05). Scale bars on the top right of each heatmap represent the degree of gene expression with red showing high expression and green indicating low expression.

To determine how parasitism affects the composition and distribution of TAGs and glycerophospholipid in *A*. *gossypii* we quantified the lipid classes in the control and parasitized aphids. Parasitism significantly influenced the TAG and PL metabolite profiles in *A*. *gossypii*. Relative difference among the lipid classes revealed significant parasitism-induced changes in the abundance of lipid species in most of the analyzed lipid classes (Fig. 3). Although parasitism did not alter the lipid class of different triacylglycerol and glycerophospholipid in *A*. *gossypii*, it increased the levels of numerous triacylglycerols and decreased the levels of many glycerophospholipid species to below the limit of detection (Fig. 4). Note that there were many unidentified lipids (ULP) that are not listed in this study.

In insects, TAGs are stored in specialized lipid droplets. Studies have shown that lipid droplets not only assume the role of passive storage, but also actively participate in fat and energy metabolism^30^. In our analysis, we found that monoacylglycerols (MGs) and diacylglycerols (DGs), which are the intermediate products in lipid synthesis, were unstable. All TAGs (Fig. 3) (TAG (18:1/18:2/18:2), 46.5-fold; TAG (18:2/18:2/18:2), 38.3-fold; TAG (18:0/18:1/18:1), 20.1-fold) differed markedly between twogroups. After parasitism, the TAGs content was significantly increased relative to the non-parasitized aphids. Consistent with this, the expression levels of several genes involved in the synthesis of TAG were up-regulated; *Ppap2a* (phosphatidic acid phosphatase type 2A) and *Gpat1* (glycerol-3-phosphate O-acyltransferase 1) in glycerolipid metabolism pathway were 7.91- and 7.80-fold higher in parasitized aphids than in non-parasitized aphids, respectively (Fig. 3D).

When analyzing the fatty acyl chains associated with triacylglycerols, we detected a marked increase in the levels of the acyl chains, C18:1 and C18:2 in major and middle TAGs of parasitized *A*. *gossypii* when compared to non-parasitized aphids (Fig. 3E,F). Interestingly, we observed that the saturated odd-numbered acyl chains (C17:0) in the middle TAG (Fig. 3F) were affected only marginally by parasitism while some very-long-chain fatty acyls (C ≥ 20) increased robustly in aphids after parasitism (eg.C20:0, C20:1, C20:2). The increased abundance of fatty acyls may have resulted from increased activity of the glycerolipid metabolism pathway. The relative peak area of quantified lipid classes in minor TAG is shown in Figure S3.

### Evaluating the Effects of *L*. *japonica* Parasitism on the *A*. *gossypii* Sphingomyelin Metabolic Pathway

**Figure 5:**
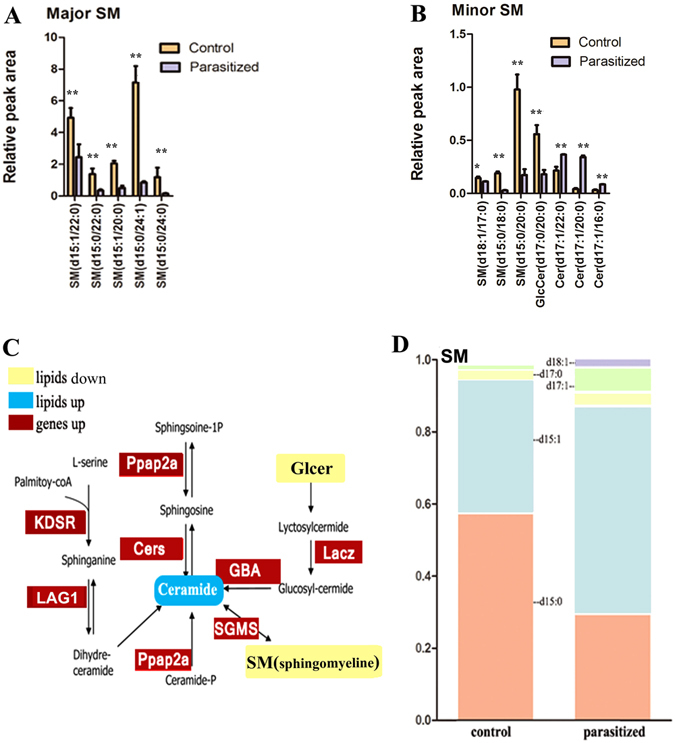
Changes in the gene expression and lipid composition in the sphingomyelin metabolism. (**A**,**B**) Quantification of the relative peak area of major sphingomyelin (**A**) and middle sphingomyelin (**B**) lipid species in parasitized and control aphids. Data are means ± SEM; n = 6/group. ***p < 0.01. (**C**) The sphingomyelin metabolism is induced in *A*. *gossypii* after exposure to cold conditions. Select sphingomyelin metabolites in the KEGG pathway, were significantly regulated in parasitized aphids. Red boxes indicate increased gene expression levels in parasitized insects and blue boxes indicate the high expression levels of sphingomyelin components. *Cers*, ceramide synthetase; *KDSR*, 3-dehydrosphinganine reductase; *SGMS*, shingomyelin synthase; *Lacz*, beta-galactosidase; *GBA*, glucosylceramidase. (**D**) Sphingomyelin base chemistry and fatty acyl chains in parasitized and control aphids. Data are means ± SEM (n = 6/group).

Analysis of sphingomyelin (SM) species revealed no changes between the parasitized and non-parasitized aphids (Fig. 5). However, numerous changes were observed in the composition of these species. All SM species (e.g., SM (15:0/24:1), 0.1-fold) and Glycosphingolipids (GlcCer) (e.g., GlcCer (d17:0/20:0), 0.3-fold) were decreased in parasitized aphids compared to non-parasitized aphids, while ceramide (e.g., cer (d17:1/20:0), 8.2-fold) was highly enriched after parasitism. Moreover, almost all genes in the SM metabolic pathway were up-regulated (Fig. 5C, Table S2). For example, *Ppap2a*, *LAG1* (acyl-CoA-dependent ceramide synthase) and cers (ceramide synthetase) were up-regulated consistent with the marked SM remodeling in response to parasitism. Analysis of SM fatty acyl chains, which mainly contains the sphingoid base, d15 and d17, revealed a high abundance of d15 and d17 indicating that parasitism induces changes in the relative proportions of the various forms of fatty acyl chains of SM (Fig. 5).

### The Glycerophospholipid Pathway is Stimulated by Parasitism

**Figure 6:**
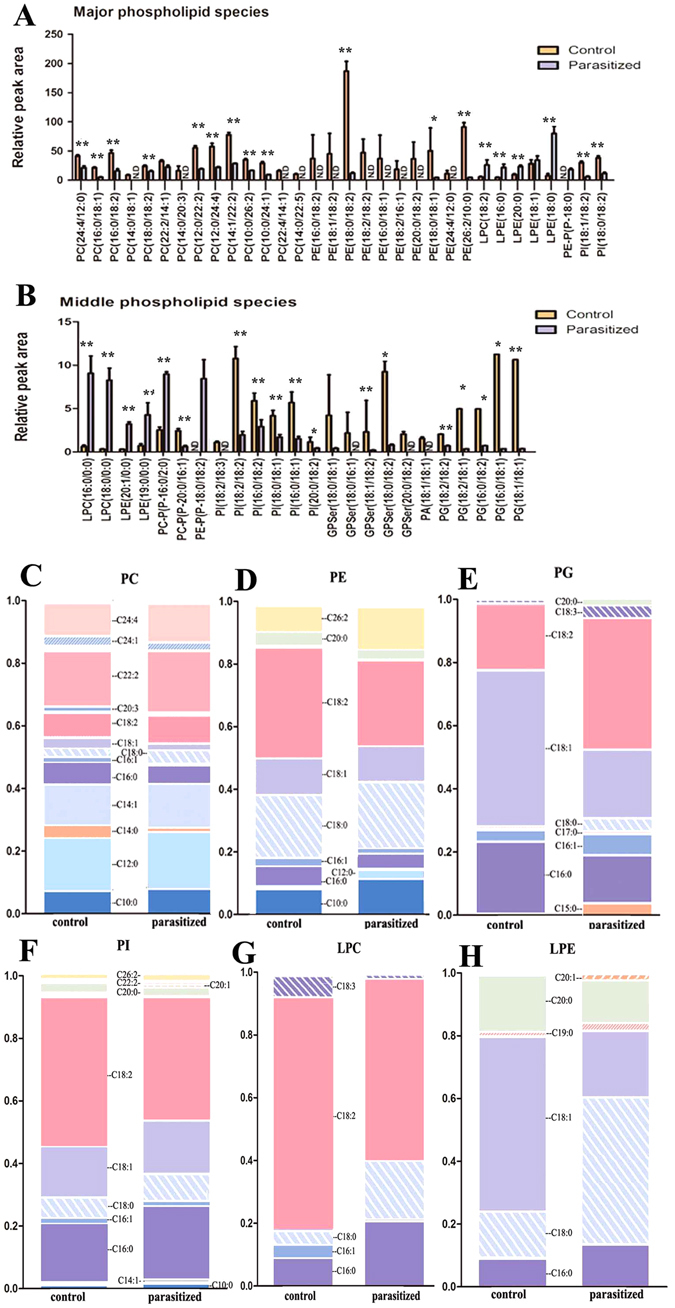
Changes in the lipid composition and fatty acyl chains of phospholipids in parasitized aphids. (**A**,**B**) Quantification of the relative peak area of major phospholipids (**A**) and middle phospholipids (**B**) in parasitized and control aphids. Data are means ± SEM; n = 6/group. *p < 0.05, **p < 0.01. N.D, not detected. (**C**–**H**) Changes in the glycerophospholipid base chemistry and fatty acyl chains in the phospholipids of parasitized and control aphids (PC, C; PE, D; PG, E; PI, F; LPC, G; LPE, H). Data are means ± SEM (n = 6/group).

Glycerophospholipid metabolism was the most significantly altered parasitized-induced gene pathway in previous studies^20, 21^. In contrast, parasitism did not alter the abundance of the glycerophospholipid classes (Fig. 6, Table S1). When compared with the control group, as many as 32 PLs (Phosphatidylcholines, PC(16:0/18:1), 0.2-fold; Phosphatidylethanolamines, PE(18/10:0), Phosphatidylinositols, PI (18:0/18:2), 0.03-fold; Phosphatidylglycerols, PG (18:2/18:1), 0.07-fold; Phosphatidylserines, GPSer(20:0/18:2), 0.05-fold) were decreased significantly in the parasitized group. In addition, a few Lysophosphatidylcholines(LPC), Lysophosphatidylethanolamines(LPE), Alkenyl-linked PC(PC-P) and Alkenyl-linked PE(PE-P) of PLs (LPC(16:0/0:0), LPE(16:0/0:0), LPC(18:2/0:0), LPE(19:0/0:0)) were increased in the parasitized group (Fig. 6). The increased levels of LPC and LPE, and the changes in their compositions indicate a marked glycerophospholipid remodeling in response to parasitism. This is also consistent with the result that parasitism leads to an increased expression of several genes involved in the synthesis and remodeling of LPC and LPE. Moreover, alkenyllinked PC and PE were only detected in the control or parasitized aphids, respectively (Fig. 6).

In this study, we detected a marked reduction in C18:2 (except PG) (Fig. 6) and only a marginal reduction in C16:0 and C16:1 (Fig. 6) in parasitized aphids. This may have resulted from the reduction in the levels of PC, PE, and PI species (Fig. 6) that contained these acyl chains. However, quantification of the base chemistry and fatty acyl chains for the individual glycerophospholipid subclasses in parasitized and non-parasitized aphids revealed a marked reduction in the PG species C18:1 (Fig. 6E) similar to the result observed in the LPE subclasses (Fig. 6H). These changes confirmed that parasitism not only changed the content of glycerophospholipid subclasses, but also resulted in the remodeling of fatty acyl chains of glycerophospholipid. This clearly indicated that when parasitized, the aphid metabolism is altered by the parasitoid so as to provide for the demands of the parasitoid itself. In addition, we demonstrated that the increased levels of LPC and LPE were accompanied by the increased levels of C16:0 and C18:0 (Fig. 6G,H). Consistent with these increases, the expression of several genes involved in the synthesis and remodeling of PC, PE, Phosphatidylinositols(PI) and PG were also increased (Fig. 6). The relative peak area of quantified lipid classes in additional middle PL and minor PL is shown in Figure S3.

**Figure 7:**
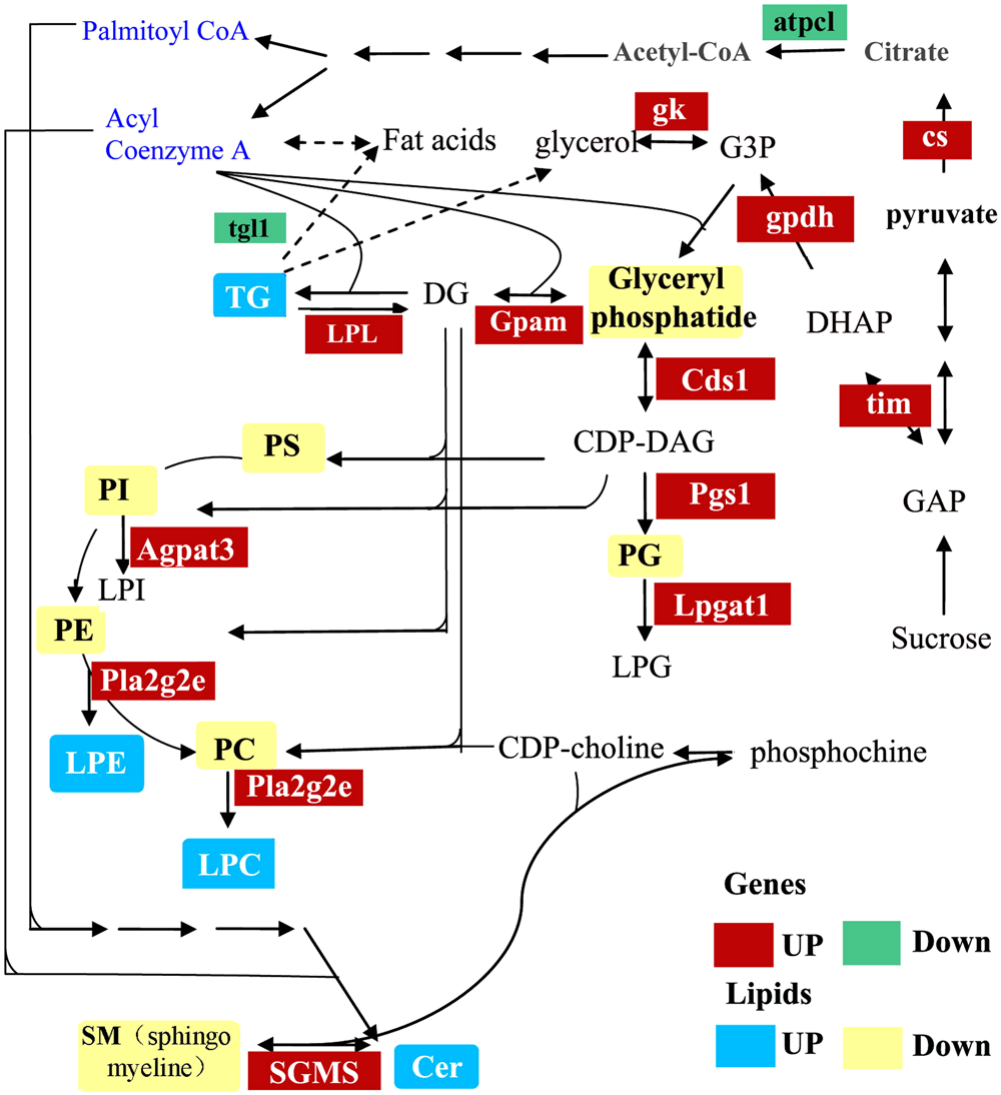
Glycerophospholipid metabolism is induced in parasitized *A*. *gossypii*. Pathway of select glycerophospholipid metabolites from the KEGG database with indications of quantified lipid classes and genes significantly regulated in parasitized aphids. Red and green colored boxes indicate increased and decreased expression levels of genes in parasitized aphids, respectively. Yellow boxes indicate decreased lipid classes and blue boxes indicate high expression of glycerophospholipids. In the fat synthesis pathway, acetyl CoA is an intermediate between the TCA cycle and the fat synthesis pathway. Besides, G3P is the intermediate between glycolysis and the fat synthesis pathway. TAGs are produced by G3P and acyl-coenzyme A; while G3P is converted to glyceraldehyde 3-phosphate (GAP) via glycolysis, and acyl CoA is converted to palmityl coenzyme A and acyl-coenzyme A. Some genes in the glycolytic pathway and TCA cycle that are believed to stimulate the phospholipid metabolic pathways are also shown. *DHAP*, dihydroxyacetone phosphate; *Lpgat1*, lysophosphatidylglycerol acyltransferase 1; *gk*, glycerol kinase.

Interestingly, the high expression of genes such as *Cds1*, *Pgs1*, *Agapt3* and *Gpam* (glycerol-3-phosphate acyltransferase) (Fig. 7, Table S3) involved in the glycerophospholipid metabolism did not contribute to the increase in PLs. Conversely, the reduction in PLs did not weaken signal transmission (Figure S1A,B). Therefore, we presume that the increases in the PL levels could be likely used by the parasitoid larvae for self-growth and tissue remodeling. Besides, some genes involved in the TCA cycle and glycolysis were also up-regulated in parasitized aphids (Figure S1).

## Discussion

Metabolites participate in multiple cellular reactions, connecting different pathways that mediate and perform several cell functions. Therefore, metabolite profiling may show changes in biological functions or phenotypes in response to parasitic^31–34^. With the exception of studies in lepidopterans^35–38^ and other model insects^39^, not much is known about how changes induced by parasitoids at the transcriptional and lipidomic levels in aphids can affect parasitoid-host relationship. In order to understand parasitism-induced changes in the composition of the different lipids classes and the expression of genes involved in the lipid metabolism we applied mass-spectrometry-based lipidomics and RNA-Seq in an attempt to provide a resource describing the molecular signature of parasitized aphids at the lipidomic and transcriptomic levels. It is known that the host aphid is killed on day 6–7 after parasitism and that adult parasitoids emerge a week later^40, 41^. We had observed that *L*. *japonica* with their 2^nd^–3^rd^ instar larval stages developing within 72 h after oviposition. Moreover, wasp mortality in highly resistant aphids typically occurs prior to 72 h^42^. So, we choose 3 d as the point time for analysis the regulation of *L*. *japonica*.

Studies reported that parasitoids can eat the host fat and modify host fat metabolism^5–9^. In this study, we analyzed 282 lipid metabolites in both parasitized and non-parasitized aphids, and found that parasitism affected the composition of lipids and remodeled them. Moreover, the contents and fatty acyl chains of PCs, PEs, PSs, PGs, SMs, LPCs, LPEs in *A*. *gossypii* were altered after parasitism. A number of studies have demonstrated the inability of parasitoid species to synthesize lipids during their life^14, 43–45^, it is evident that it must obtain exogenous sources to complete life cycle. Our data showed a sharp increase in the TAG levels and the downregulated of the *tgl1* in *A*. *gossypii* after parasitism by *L*. *japonica*. In all eukaryotic organisms, TAG synthesis and mobilization play important roles in energy homeostasis^46^. The main function of fat cells is stored energy in the form of triacylglycerol, and released to supply to the body when the body needs, The higher levels of TAGs could likely meet the demand of lipid and energy of *L*. *japonica* that is required for flight, mating activities, and oviposition^47^ in the adult stage.

Lipid reserves play a key role in both survival and reproduction^48^. Sphingolipids comprise a complex and ubiquitous class of membrane lipids^49^, which are recognized as key regulators of cell cycle progression as well as growth and cellular metabolism^50^. In our data, among the major lipid classes analyzed, sphingolipids were more abundant in the non-parasitized group (e.g., d15:0/24:1, except Cer) although we cannot exclude direct germline effects because gonads were included in the parasitized aphid samples. SM metabolites have been implicated in brain development and neuronal function in *Drosophila* spp and sphingolipid metabolism was reported to elicit apoptosis-associated reproductive defects^51–53^. Additionally, we found high levels of Cer after parasitism suggesting a potential link between parasitism-associated biochemical alterations and the direct cytopathic effect observed in the host system^54^. Ceramides and other sheath phospholipids act as second messengers, regulating the action of many target proteins by inducing cascade amplification of enzyme activity. Cer may play a critical role in *L*. *japonica* development although these are minor PL species. Alternatively, the reduced levels of SM species and GlcCer could be necessary for aphid growth and reproduction.

Our data demonstrated that a significant remodeling of glycerolipid and glycerophospholipid in response to parasitism. In the *L*. *japonica* – *A*. *gossypii* association, parasitism induced differential changes in the different subclasses of acyl chains and subspecies of TAG and PL. This highly specific change may be a selective regulation by *L*. *japonica*; alternatively, it could be the coping strategy of *A*. *gossypii* when parasitized. The highly selective remodeling of lipids subspecies is likely to have significant functional implications on *L*. *japonica* growth and reproduction.

Host development is regulated by factors that are heredity^55, 56^ or are injected by the parasitoids during oviposition^57^. Based on the effects of parasitism on lipidomics, we focused our analyses on glycerolipid, glycerophospholipid and SM metabolic pathways in parasitized aphids. Our study indicated that the expression of a large number of genes in these pathways was induced significantly in parasitized aphids. An interesting finding from our study is the down-regulation of *tgl1* mRNA (2.7-fold) in parasitized aphids, *tgl* plays a negative role in the TAG metabolism, but the other way of TAG metabolism was induced, resulted from the upregulated of *LPL*. In addition, *Gpam* is increased 7.8-fold in parasitized aphids, this indicated that the increased TAG were mainly used to synthesize DG which may contributed to synthesize Lysophosphatidyl. Moreover, *Agpat3*, *Cds1*, *Lpgat1*, *Pgs1* and *Pla2g2e* that regulate Lysophosphatidyl phospholipid synthesis genes were induced after parasitism for 3 days. Lysophosphatidyl display the role of neurotoxicity and can make cells dissolve in low dose^58^. Parasitoids regulate the physiological milieu of the host to facilitate utilization of host nutrients by the parasitoid^31, 32, 59, 60^, it chould be seen that aphids were controled to lysis cells and emit energy after parasitized by regulating the target genes which contributed to synthesize Lysophosphatidyl. The biological and physiological importance of the up- and down-regulation of these genes should be investigated further using knockout models.

Major metabolic pathways associated with sugar and lipid metabolism are typically highly conserved across taxa^61^. Studies had reported that all lipid metabolism related genes in glycolysis pathway were expressed in high levels in parasitized aphids^20, 21^. Furthermore, parasitism was also shown to enhance glycolysis and the TCA cycle^62, 63^. Our data demonstrated that genes involved in TCA cycle and glycolysis pathway had increased expression. Typically, *A*. *gossypii* feeds on plant phloem, an abundant source of carbon and energy. Sucrose carbons in aphid tissues are mainly converted into lipids^64^. Previous genomic analysis indicated that TAG is synthesized by the glycerol 3-phosphate (G3P) pathway, which is the sole pathway for TAGs synthesis in *Rhodnius prolixus* ^20^, and when *A*. *gossypii* was parasitized by *Praon volucre*, glycolysis was galvanized, likely to favor polyols biosynthesis^65^. Our data showed that some genes involved in the TCA cycle and glycolysis (e.g., *atpcl*, *gk*, *gpdh* (glycerol-3-phosphate dehydrogenase) and *tim* (triosephosphate isomerase)) were up-regulated consistent with previous reports^21^. We presume that glycerophospholipid metabolism was accompanied by TCA and glycolysis pathway, and the induced TCA and glycolysis pathway was necessary for the synthesize of TAGs and PLs. What’ more, more metabolic changes may occur during the parasitized stage to adapt to the changing environment of aphids.

Our study has provided insights on the TAG and PL diversity and biochemistry during parasitism. Our integrated results indicate that parasitism may activate the transcriptional program of glycerolipid and glycerophospholipid metabolism in aphids, enhance the expression of genes involved in SM metabolism as well as the TCA cycle and glycolysis pathway and may be accompanied by remodeling of the triacylglycerol and glycerophospholipid composition. These changes are highly specific to this species. The biochemical information we described here will help future studies investigating the regulation of host lipid metabolism by parasitoids.

## Conclusion

In this study, we have reported changes in the lipidomic and transcriptional profiles of parasitized cotton-melon aphids for the first time. Our comprehensive analysis shows that parasitism can markedly enhance the expression of genes involved in the glycerolipids and glycerophospholipids pathways and also results in the remodeling of triacylglycerol and glycerophospholipids. These results show that *L*. *japonica* can influence the hosts’ nutritional physiology to benefit its own growth while deterring *A*. *gossypii* development. These data may serve as a valuable resource for studies on parasitoid-insect interactions. Such a comprehensive study on the lipidomics of parasitoids and its host species would help us not only to determine their dietary requirements but also help improve parasitoid mass rearing for use in biological control programs.

## Electronic supplementary material

Supplementary Information

Table S1

Table S2
