# Supplementary material for: Lipidomics and RNA-Seq Study of Lipid Regulation in Aphis gossypii parasitized by Lysiphlebia japonica
Source: Sci Rep. 2017 May 2;7:1364. doi: 10.1038/s41598-017-01546-1 (PMC5431011; doi:10.1038/s41598-017-01546-1)
Supplement: Supplementary file 1 — Supplementary Information [file 41598_2017_1546_MOESM1_ESM.pdf]

# Supplementary Information

## Lipidomics and RNA-Seq Study of Lipid Regulation in *Aphis gossypii* parasitized by *Lysiphlebia japonica*

Gao Xueke, Zhang Shuai, Luo Junyu, Lü Limin, Zhang LiJuan, Cui Jinjie *

*(Institute of Cotton Research, Chinese Academy of Agricultural Sciences / State Key Laboratory of Cotton Biology, Anyang, Henan 455000, China)*

*Correspondence and requests for materials should be addressed to J.-J.C. (cuijinjie@126.com).

This Supplementary Information contains:

Supplementary Figure Legends

Supplementary Figures S1-S3, Table S3

# Supplementary Figure Legends

**Figure S1.Parasitied Exposure Induces Gene Expression Involved in Metabolism.** Heatmap representation of changes of genes expression that significantly enriched metabolic KEGG pathways with parasitized (Antigen processing and presentation, A; MAPK signaling pathway ,B; Citrate cycle (TCA cycle), C; Glycolysis, D). The log10 expression values for each sample were clustered and uniformed in rows.

**Figure S2. Loading Scatter Plot and S-plot of OPLS-DA model obtained from D and F samples (POS and NEG).**

Loading Scatter Plot of OPLS-DA model obtained from D and F(POS). (B) S-plot of OPLS-DA model obtained from D and F(POS). (C) Loading Scatter Plot of OPLS-DA model obtained from D and F(NEG). (D S-plot of OPLS-DA model obtained from D and F(NEG). Ends of the loading Scatter Plot and S-plot of OPLS-DA model were potential differences markers. The farther the distance from the center on behalf of the degree of difference.

**Figure S3**. **Changes in lipid composition of PL and TG under parasitized exposure.**

(A-E) The relative peak area of quantified lipid classes in minor TG (A), middle PL(B) and minor PL (C-E) of parasitized-treated and control group. Data are presented as means + SEM; n = 6 for both groups. Significance level: *$p < 0.05$,**$p < 0.01$. N.D., not detected.

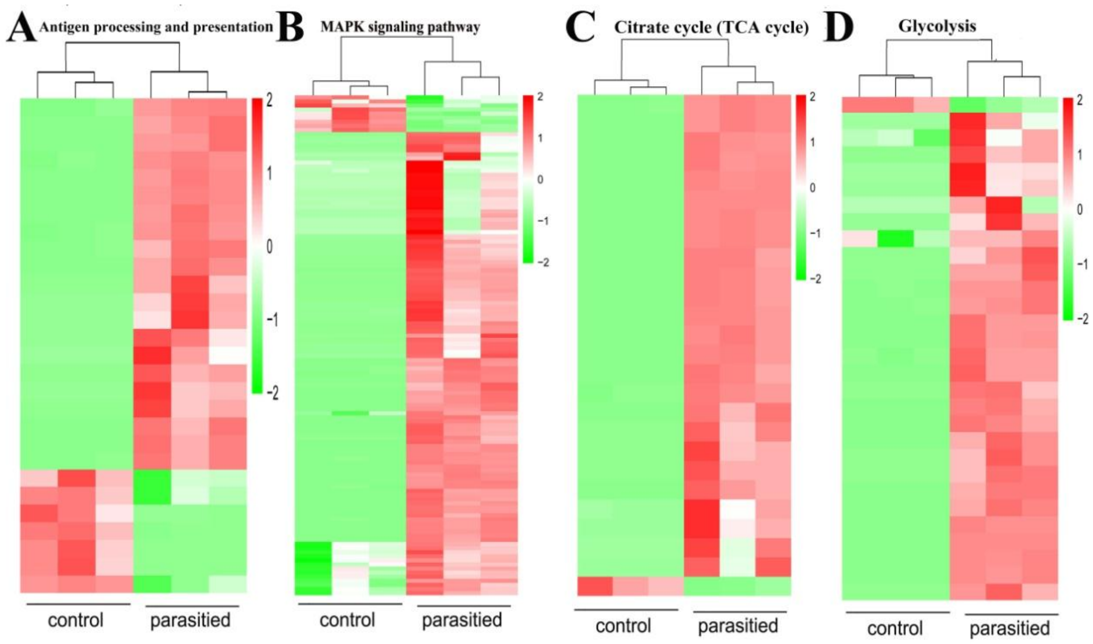
A Antigen processing and presentation
B MAPK signaling pathway
C Citrate cycle (TCA cycle)
D Glycolysis
2
1
0
-1
-2
control
parasitied
control
parasitied
control
parasitied
control
parasitied

Figures S1

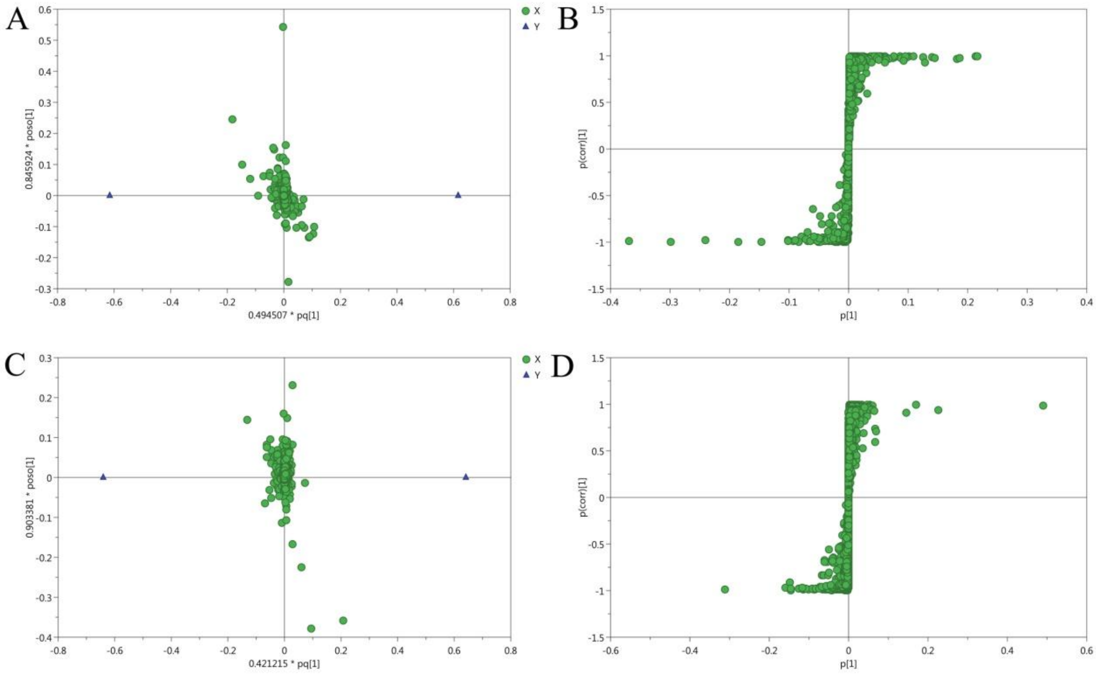
A
B
C
D
X
Y
0.845924 * poso[1]
0.494507 * pq[1]
p(corr)[1]
p[1]
0.903381 * poso[1]
0.421215 * pq[1]

Figures S2

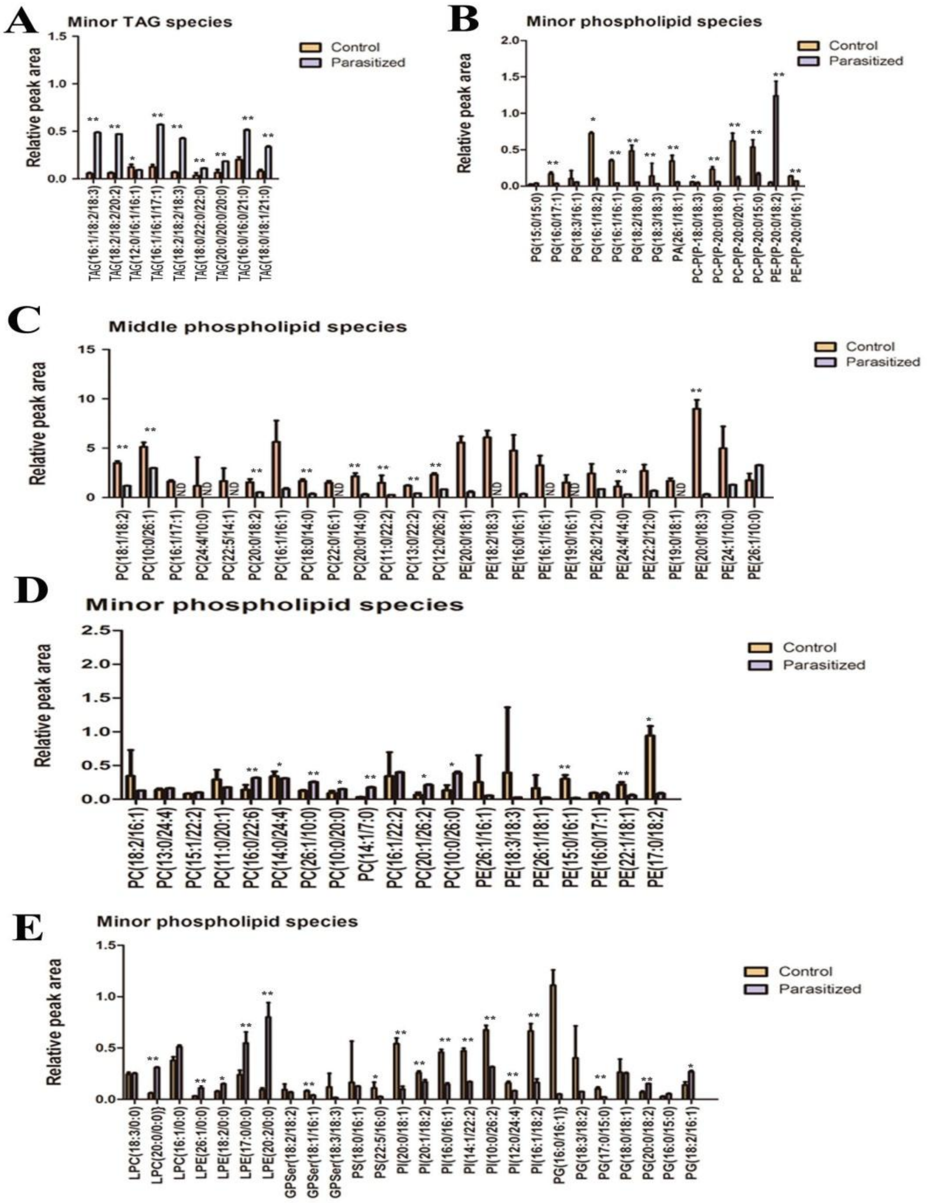
A
Minor TAG species
Control
Parasitized
Relative peak area
TAG(16:1/18:2/18:3)
TAG(18:2/18:2/20:2)
TAG(12:0/16:1/16:1)
TAG(16:1/16:1/17:1)
TAG(18:2/18:2/18:3)
TAG(18:0/22:0/22:0)
TAG(20:0/20:0/20:0)
TAG(16:0/16:0/21:0)
TAG(18:0/18:1/21:0)
B
Minor phospholipid species
Control
Parasitized
Relative peak area
PG(15:0/15:0)
PG(16:0/17:1)
PG(18:3/16:1)
PG(16:1/18:2)
PG(16:1/16:1)
PG(18:2/18:0)
PG(18:3/18:3)
PA(26:1/18:1)
PC-P(P-18:0/18:3)
PC-P(P-20:0/18:0)
PC-P(P-20:0/20:1)
PC-P(P-20:0/15:0)
PE-P(P-20:0/18:2)
PE-P(P-20:0/16:1)
C
Middle phospholipid species
Control
Parasitized
Relative peak area
PC(18:1/18:2)
PC(10:0/26:1)
PC(16:1/17:1)
PC(24:4/10:0)
PC(22:5/14:1)
PC(20:0/18:2)
PC(16:1/16:1)
PC(18:0/14:0)
PC(22:0/16:1)
PC(20:0/14:0)
PC(11:0/22:2)
PC(13:0/22:2)
PC(12:0/26:2)
PE(20:0/18:1)
PE(18:2/18:3)
PE(16:0/16:1)
PE(16:1/16:1)
PE(19:0/16:1)
PE(26:2/12:0)
PE(24:4/14:0)
PE(22:2/12:0)
PE(19:0/18:1)
PE(20:0/18:3)
PE(24:1/10:0)
PE(26:1/10:0)
D
Minor phospholipid species
Control
Parasitized
Relative peak area
PC(18:2/16:1)
PC(13:0/24:4)
PC(15:1/22:2)
PC(11:0/20:1)
PC(16:0/22:6)
PC(14:0/24:4)
PC(26:1/10:0)
PC(10:0/20:0)
PC(14:1/7:0)
PC(16:1/22:2)
PC(20:1/26:2)
PC(10:0/26:0)
PE(26:1/16:1)
PE(18:3/18:3)
PE(26:1/18:1)
PE(15:0/16:1)
PE(16:0/17:1)
PE(22:1/18:1)
PE(17:0/18:2)
E
Minor phospholipid species
Control
Parasitized
Relative peak area
LPC(18:3/0:0)
LPC(20:0/0:0)
LPC(16:1/0:0)
LPE(26:1/0:0)
LPE(18:2/0:0)
LPE(17:0/0:0)
LPE(20:2/0:0)
GPSer(18:2/18:2)
GPSer(18:1/16:1)
GPSer(18:3/18:3)
PS(18:0/16:1)
PS(22:5/16:0)
PI(20:0/18:1)
PI(20:1/18:2)
PI(16:0/16:1)
PI(14:1/22:2)
PI(10:0/26:2)
PI(12:0/24:4)
PI(16:1/18:2)
PG(16:0/16:1)
PG(18:3/18:2)
PG(17:0/15:0)
PG(18:0/18:1)
PG(20:0/18:2)
PG(16:0/15:0)
PG(18:2/16:1)

Figures S3

Table S3 : Expression of selected genes related to figure 5 and figure 7.

| gene | control | parasitized | $Log_2$FD(parasitized /control) | P-value |
|---|---|---|---|---|
| psd | 0 | 17.68 | 7.183274 | 3.70E-20 |
| agpat1 | 0 | 6.43 | 2.72858 | 0.004088 |
| atpcl | 12670.38 | 167.07 | -1.12585 | 9.62E-06 |
| cs | 2.58 | 498.16 | 10.61391 | 6.81E-68 |
| pgs1 | 0 | 3.49 | 4.969676 | 1.75E-08 |
| pla2g2e | 0 | 15.24 | 7.190428 | 8.38E-21 |
| cds1 | 0 | 6.05 | 5.323815 | 1.42E-09 |
| agpat3 | 0 | 48.98 | 8.792222 | 5.35E-36 |
| LPL | 412.86 | 24.02 | 1.0943 | 5.99E-06 |
| dgat1 | 0.75 | 18.67 | 6.544471 | 2.95E-16 |
| dgk | 129.91 | 10.3 | 1.46576 | 0.000326 |
| gpat1 | 0 | 14.84 | 7.391325 | 7.72E-24 |
| plsc | 0 | 48.98 | 8.792222 | 5.35E-36 |
| agk | 0 | 18.48 | 7.325134 | 2.29E-21 |
| Ppap2a | 0 | 7.91 | 5.82694 | 9.34E-12 |
| kdsr | 0.75 | 14.89 | 6.494432 | 1.05E-16 |
| LAG1 | 0 | 23.9 | 7.945567 | 4.25E-28 |
| cers | 0 | 14.87 | 6.91458 | 4.36E-18 |
| SGMs | 226.02 | 18.79 | 1.604574 | 1.78E-05 |
| GBA | 0 | 2.58 | 3.759893 | 6.10E-05 |
| Lacz | 0 | 62.24 | 8.235971 | 3.23E-26 |
| gk | 0 | 31.49 | 7.664303 | 8.49E-23 |
| gpdh | 0 | 111.76 | 9.799776 | 6.14E-49 |
| tim | 1.82 | 301.53 | 10.49078 | 2.23E-67 |
| pgam2 | 0 | 192.26 | 10.5611 | 4.81E-60 |
| pgam5 | 0 | 12.94 | 6.871849 | 3.43E-18 |
| lpgat1 | 0 | 11.58 | 6.941368 | 1.24E-19 |
| tgl1 | 326.19 | 0.42 | -2.67963 | 2.00E-24 |
| tgl2 | 187.68 | 16.44 | 1.661247 | 6.00E-17 |
| tgl3 | 349.37 | 105.68 | 3.460447 | 3.00E-67 |
